# Ethical leadership and eudaimonic well-being at work: the roles of organizational identification and caring ethical climate

**DOI:** 10.3389/fpsyg.2026.1903879

**Published:** 2026-07-29

**Authors:** Zhixiao Ye, Jiaming Hu, MyeongCheol Choi, WonGyu Lee

**Affiliations:** 1 Department of Property Management, Zhejiang Shuren University, Hangzhou, China; 2 Department of Business Management, Gachon University, Seongnam, Republic of Korea; 3 Department of Intellectual Property Convergence, Gyeongsang National University, Jinju, Republic of Korea

**Keywords:** caring ethical climate, eudaimonic well-being, organizational identification, organizational psychology, personal growth, positive relations with others

## Abstract

**Introduction:**

Ethical leadership has been widely examined as an important antecedent of employee attitudes and behaviors, yet less is known about how it contributes to employees’ eudaimonic well-being at work. Drawing on ethical leadership theory and social identity theory, this study examines how ethical leadership is associated with two dimensions of workplace eudaimonic well-being, namely positive relations with others and personal growth. It further investigates the mediating role of organizational identification and the moderating role of caring ethical climate.

**Methods:**

Data were collected through a two-wave time-lagged survey of 310 employees from project and property management enterprises in China. Confirmatory factor analysis, structural equation modeling, bootstrap mediation analysis, and hierarchical regression analysis were used to test the proposed model.

**Results:**

The results showed that ethical leadership was positively associated with organizational identification, positive relations with others, and personal growth. Organizational identification partially mediated the relationships between ethical leadership and both well-being dimensions. Caring ethical climate strengthened the associations between ethical leadership and organizational identification, positive relations with others, and personal growth.

**Discussion:**

These findings suggest that ethical leadership is associated with employees’ relational and developmental well-being not only through organizational identification but also within a caring ethical work climate. This study contributes to organizational psychology research by clarifying the identity-based mechanism and ethical climate boundary condition through which ethical leadership supports eudaimonic well-being at work.

## Introduction

Employee psychological well-being is an important issue in organizational behavior and behavioral science (Kia et al., 2019). As organizational uncertainty, service pressure, and the intensity of interpersonal interactions continue to increase, employees are expected not only to complete their work tasks but also to maintain positive interpersonal relationships, experience personal growth, and sustain sound psychological functioning at work (Liu et al., 2023).

Psychological well-being does not merely refer to the reduction of negative emotions; it also reflects positive states related to relationship quality, self-development, meaning, and adaptive capacity. In recent years, research on employee well-being has gradually moved beyond a narrow focus on job satisfaction or emotional experience toward a more comprehensive understanding of psychological functioning, social relationships, and self-realization (Martela, 2025). This issue is particularly salient in service-oriented organizations, where employees frequently face customer demands, internal coordination, performance pressure, and emotional labor. Their psychological state can directly influence service quality, organizational commitment, and sustainable performance (Jaswal et al., 2024). Therefore, examining which organizational factors can promote employee psychological well-being has important theoretical and practical significance.

Among the organizational factors that influence employees’ psychological well-being, leadership behavior plays a critical role (Brown et al., 2005). Recent studies on value-based leadership also suggest that leadership styles grounded in authenticity, ethics, service, and purpose may contribute to ethical workplace cultures, employee engagement, organizational resilience, workplace well-being, and ethics (Zhu, 2025a, 2025b). These studies further indicate that morally grounded leadership behaviors are relevant for understanding employees’ psychological and ethical work experiences. Ethical leadership emphasizes leaders’ fairness, honesty, care, and moral consistency in decision-making, communication, reward and punishment, and daily management practices. Compared with general leadership behaviors, ethical leadership not only focuses on task accomplishment but also attaches importance to moral standards and respect for employees in the management process. Ethical leadership theory suggests that leaders provide employees with observable and learnable standards of ethical conduct through moral role modeling, two-way communication, and fair reward and punishment systems (Brown and Treviño, 2006). Based on social cognitive theory, leaders’ behaviors serve as an important source of information through which employees understand organizational norms, form behavioral expectations, and adjust their psychological responses (Bandura, 2001). Therefore, when employees perceive that their leaders treat them fairly, listen attentively, and uphold ethical standards, they are more likely to develop a sense of security, trust, and respect, thereby establishing positive interpersonal relationships at work and experiencing stronger personal growth.

However, existing research on ethical leadership and employee well-being still leaves room for further development. Prior studies have mainly focused on the effects of ethical leadership on job satisfaction, organizational commitment, work engagement, organizational citizenship behavior, or the reduction of unethical behavior. However, relatively limited attention has been paid to the internal dimensions of psychological well-being. A relevant meta-analysis indicates that ethical leadership can influence a wide range of positive employee attitudes and behavioral outcomes, but the specific mechanisms underlying different psychological outcomes require further clarification (Bedi et al., 2016). Psychological well-being is a multidimensional construct, in which positive relations with others and personal growth are two representative dimensions. Positive relations with others reflect whether employees can establish trust, support, and cooperative relationships at work, whereas personal growth reflects whether employees experience continuous learning, self-development, and capability enhancement (Ryff and Keyes, 1995; Ryff and Singer, 1998). These two dimensions are particularly important for project and property management enterprises, because employees in this sector frequently deal with relationships among residents, customers, contractors, and internal departments, while also needing to improve communication, coordination, and problem-solving abilities in complex service contexts.

In addition to directly influencing employees’ psychological well-being, ethical leadership may also exert its effects through organizational identification. Organizational identification refers to the extent to which employees define themselves as members of the organization and develop a psychological connection with its values, goals, and identity (Ashforth and Mael, 1989). In the Chinese organizational context, leadership behavior is particularly important for shaping employees’ understanding of organizational values and their psychological attachment to the organization. Employees often regard supervisors as representatives of the organization and interpret leaders’ fairness, honesty, care, and moral consistency as signals of whether the organization is trustworthy and value-consistent. This is especially relevant in project and property management enterprises, where employees frequently face customer complaints, interpersonal tensions, service pressure, and cross-departmental coordination demands. When leaders demonstrate fairness, honesty, and care, employees are more likely to perceive the organization as trustworthy and worthy of identification, thereby strengthening their sense of belonging and value congruence. Higher organizational identification enables employees to internalize organizational goals as their own and to display greater willingness to cooperate, emotional involvement, and positive psychological states at work. Existing research has also shown that organizational identification is closely related to employee health and well-being and may serve as an important mechanism through which the organizational environment shapes employees’ psychological well-being (Hameed et al., 2022; Greco et al., 2022). Therefore, organizational identification may represent an important psychological mechanism linking ethical leadership to employees’ psychological well-being. In other words, ethical leadership may not only directly influence employees’ positive relations with others and personal growth but also further promote psychological well-being by enhancing employees’ identification with the organization.

At the same time, the effects of ethical leadership do not occur in a vacuum. Employees’ interpretations of and responses to leadership behavior often depend on the broader organizational ethical environment. Ethical climate refers to organizational members’ shared perceptions of ethical norms, practices, and procedures. Among its dimensions, a caring ethical climate emphasizes concern for others’ interests, mutual support, and collective well-being in organizational decision-making and actions (Victor and Cullen, 1988). In such a climate, employees are more likely to perceive organizational benevolence, support, and fairness, and they may also interpret leaders’ ethical behavior as part of the organization’s broader value system. Prior research suggests that ethical leadership and ethical climate can jointly shape the organizational ethical environment and further influence employees’ attitudes, behaviors, and well-being (Kuenzi et al., 2020; Paralta et al., 2023). In addition, recent research has found that a caring ethical climate contributes to employees’ sense of meaning and affective well-being (Pandey et al., 2024). Therefore, a caring ethical climate is not only a manifestation of the organizational ethical environment but also an important boundary condition that determines whether ethical leadership can be effectively translated into positive psychological outcomes for employees.

Based on the above discussion, this study develops a model integrating mediation and moderation effects, using employees from project and property management enterprises in China as the research context. This topic is particularly important in China because employees in this sector frequently perform boundary-spanning service work and are required to handle customer complaints, community services, emergencies, cross-departmental coordination, and performance demands. These conditions make fairness, care, responsibility, and ethical judgment especially important in everyday management. In such a context, ethical leadership may help employees perceive the organization as fair, trustworthy, and supportive, thereby strengthening their organizational identification and eudaimonic well-being at work. This study goes beyond prior research by focusing on positive relations with others and personal growth as two specific dimensions of workplace eudaimonic well-being, rather than treating employee well-being as a broad general outcome. It also examines organizational identification as an identity-based mechanism linking ethical leadership to these well-being dimensions and considers caring ethical climate as an ethical contextual boundary condition. Specifically, this study examines whether ethical leadership is positively associated with positive relations with others and personal growth. It further investigates the mediating role of organizational identification in these relationships and analyzes whether caring ethical climate moderates the relationships between ethical leadership and organizational identification, positive relations with others, and personal growth. Methodologically, this study uses a two-wave time-lagged survey of 310 employees from project and property management enterprises in China and tests the proposed model using confirmatory factor analysis, structural equation modeling, bootstrap mediation analysis, and hierarchical regression analysis.

## Theoretical background and hypotheses

### Ethical leadership

Ethical leadership refers to leaders’ demonstration of normatively appropriate conduct through personal actions and interpersonal relationships, as well as their promotion of such conduct among followers through communication, reinforcement, and decision-making (Al Halbusi et al., 2023). It emphasizes not only leaders’ personal moral qualities but also their active role in guiding employees’ ethical understanding and behavior in organizations. The core characteristics of ethical leadership include fair decision-making, honesty, concern for employees, consistent ethical standards, ethical role modeling, and the use of rewards and discipline to encourage appropriate behavior (Kim et al., 2023). Through these behaviors, ethical leaders help employees understand what kinds of conduct are acceptable and valued in the workplace (Saleem et al., 2024). From the perspective of social learning theory, employees learn appropriate attitudes and behaviors by observing leaders who are perceived as credible, legitimate, and morally consistent (Treviño et al., 2003).

Although ethical leadership is related to other positive leadership styles, it has a distinct theoretical focus. Transformational leadership emphasizes vision, inspiration, and change-oriented motivation, authentic leadership focuses on self-awareness and relational transparency, and servant leadership emphasizes serving followers’ needs and supporting their development (Islam et al., 2024a). By contrast, ethical leadership places greater emphasis on leaders’ fairness, moral role modeling, ethical communication, and the reinforcement of ethical standards (Mayer et al., 2012). This makes ethical leadership particularly suitable for the present study, because employees in project and property management enterprises frequently face customer complaints, interpersonal tensions, and coordination demands, where fairness, respect, care, and ethical judgment are central to how employees interpret the organization and their work experiences. However, prior studies have mainly examined ethical leadership in relation to general employee attitudes and behaviors, while less attention has been paid to its association with specific dimensions of workplace eudaimonic well-being, such as positive relations with others and personal growth (Li et al., 2022). This study therefore examines ethical leadership as a key leadership factor that may be associated with employees’ organizational identification, positive relations with others, and personal growth.

### Psychological well-being

Psychological well-being refers to individuals’ positive psychological functioning and the realization of their personal potential, rather than merely the absence of negative emotions or psychological distress (Soren and Ryff, 2023). From a eudaimonic perspective, psychological well-being emphasizes whether individuals can develop themselves, build meaningful relationships, pursue purpose, and function effectively in social and organizational life (Ryff, 2014). In the workplace, psychological well-being is particularly important because employees’ psychological functioning can influence their work attitudes, interpersonal interactions, and long-term development (Carmona-Rincón et al., 2023).

Among the dimensions of psychological well-being, this study focuses on positive relations with others and personal growth. Positive relations with others refer to employees’ ability to build warm, trusting, and supportive relationships in the workplace, while personal growth refers to employees’ perception that they are continuously learning, developing, and realizing their potential (Ryff and Singer, 2008). These two dimensions are especially relevant to project and property management enterprises, where employees frequently engage in service interactions, customer communication, and cross-departmental coordination. In such contexts, maintaining high-quality interpersonal relationships and experiencing personal growth are important indicators of employees’ psychological well-being.

### Organizational identification

Organizational identification refers to the extent to which employees define themselves in terms of their membership in an organization and perceive a sense of oneness with the organization (Hameed et al., 2022). It reflects a psychological connection between the individual and the organization, through which employees integrate organizational values, goals, and identity into their self-concept (Dutton et al., 1994; Edwards and Peccei, 2007). When employees strongly identify with their organization, they are more likely to perceive organizational success and failure as personally meaningful and to develop a stronger sense of belonging (Salin et al., 2023).

Organizational identification is rooted in social identity theory, which suggests that individuals partly define themselves through their membership in social groups (Li et al., 2025). In organizational settings, this means that employees who identify with the organization tend to internalize organizational values and act in ways that support organizational goals (Mael and Ashforth, 1992). Prior research has shown that organizational identification is closely related to employees’ attitudes and behaviors, including commitment, satisfaction, cooperation, and performance (He and Brown, 2013; Lee et al., 2015). Therefore, organizational identification can be regarded as an important psychological mechanism through which leadership and organizational contexts influence employees’ positive relations with others and personal growth.

### Ethical leadership and psychological well-being dimensions

Ethical leadership may promote employees’ psychological well-being by creating a fair, respectful, and morally supportive work environment (Islam et al., 2024b). Ethical leaders provide employees with clear ethical standards, make decisions in a fair and consistent manner, and show concern for employees’ needs and development (Chen et al., 2026). These behaviors can reduce uncertainty in daily work and help employees feel respected, valued, and psychologically secure. Such a work context is particularly important for positive relations with others, because employees who experience fair and respectful leadership are more likely to develop trust, engage in constructive communication, and build supportive relationships with colleagues and other organizational members (Mayer et al., 2012).

Ethical leadership may also contribute to employees’ personal growth. By emphasizing fairness, responsibility, and care, ethical leaders can provide employees with a work environment that supports learning, self-reflection, and development. Employees who perceive their leaders as ethical are more likely to feel encouraged to take on challenges, improve their abilities, and pursue meaningful work goals (Zhang et al., 2025). Recent research suggests that ethical leadership can enhance employees’ well-being by supporting emotion management and creating a psychologically supportive work context (Saleem et al., 2024). In addition, ethical leaders can strengthen employees’ motivation and engagement by building trust and encouraging harmonious work passion (Islam et al., 2024a,b). Thus, ethical leadership is likely to help employees experience continuous development and personal growth.

*H1a*: Ethical leadership has a positive effect on positive relations with others.

*H1b*: Ethical leadership has a positive effect on personal growth.

### Ethical leadership and organizational identification

Ethical leadership is likely to enhance employees’ organizational identification because employees often interpret leaders’ behavior as a signal of organizational values and norms. When leaders act with fairness, integrity, responsibility, and concern for employees, they communicate that the organization is trustworthy, respectful, and value-consistent. Such signals help employees perceive a stronger fit between their personal values and organizational values, thereby strengthening their sense of belonging and psychological attachment to the organization (Lorenz, 2025). From a social identity perspective, employees are more likely to identify with an organization when they perceive it as morally legitimate and capable of enhancing their self-concept. Recent studies have also shown that ethical leadership can strengthen organizational identity by encouraging employee voice, trust, and value alignment (Hosseini and Ferreira, 2023). Moreover, ethical leaders may become more influential when employees perceive them as representatives of the organization, because leader behavior is then more likely to be generalized to the organization as a whole (Costa et al., 2022). In this way, ethical leadership can help employees feel valued, respected, and psychologically connected to organizational values. Consistent with this reasoning, recent research suggests that ethical leadership can promote stronger organizational identification, which in turn contributes to employees’ positive organizational behaviors (Kim and Kim, 2026). Therefore, this study proposes the following hypothesis:

*H2*: Ethical leadership has a significant positive effect on organizational identification.

### Organizational identification and psychological well-being dimensions

Organizational identification is likely to promote employees’ positive relations with others because it strengthens employees’ sense of belonging, shared membership, and psychological connection with the organization (Ma et al., 2022). Employees who strongly identify with their organization tend to perceive other organizational members as part of a shared collective, which can encourage cooperation, mutual support, and constructive interpersonal interaction. In this sense, organizational identification may help employees build more trusting and supportive relationships at work (Teresi et al., 2024). Recent studies have shown that organizational identification is closely associated with employees’ well-being and positive organizational outcomes, suggesting that identification can function as an important psychological resource in the workplace (De Giorgio et al., 2023). In addition, research on workplace social networks indicates that employees’ relationship ties are closely linked to organizational identification and well-being, further suggesting that identification and interpersonal functioning are mutually reinforcing in organizational settings (Yue et al., 2025).

Organizational identification may also contribute to employees’ personal growth. When employees identify strongly with their organization, they are more likely to regard organizational goals and values as personally meaningful (Marini et al., 2025). This psychological connection can motivate employees to invest more effort in their work, accept developmental challenges, and seek opportunities for learning and self-improvement. A strong sense of identification may also enhance employees’ self-concept by linking personal development with organizational membership and collective success (Salin et al., 2023). Recent review evidence suggests that organizational identification helps employees engage intellectually and emotionally with the organization, which can support personal growth and alignment with organizational values (Divya and Christopher, 2024). Therefore, employees with stronger organizational identification are more likely to experience continuous development and personal growth in the workplace. Based on this reasoning, this study proposes the following hypothesis:

*H3a*: Organizational identification has a significant positive effect on positive relations with others.

*H3b*: Organizational identification has a significant positive effect on personal growth.

### The mediating role of organizational identification

Ethical leadership may influence employees’ positive relations with others and personal growth through organizational identification (Jamil and Fatima, 2023). Ethical leaders communicate fairness, integrity, and care through their daily decisions and interpersonal interactions, which helps employees perceive the organization as trustworthy and value-consistent (Burhan et al., 2023). When employees develop stronger organizational identification, they are more likely to regard themselves as part of the organizational collective and to interpret organizational goals and values as personally meaningful. In this process, organizational identification can transform the ethical signals provided by leaders into employees’ positive psychological and relational outcomes. Prior research has shown that ethical leadership can affect employees’ attitudes and behaviors through identification-based mechanisms, suggesting that organizational identification is an important psychological pathway linking ethical leadership to employee outcomes (Qian and Jian, 2020; Wu, 2021).

Specifically, employees who identify strongly with their organization are more likely to view colleagues as members of the same collective, which can strengthen trust, cooperation, and supportive interpersonal interactions (Ete et al., 2022). Therefore, organizational identification may explain why ethical leadership promotes positive relations with others. In addition, organizational identification can help employees attach personal meaning to organizational membership and become more willing to invest effort in learning, self-improvement, and developmental challenges (Rubab et al., 2023). In this way, organizational identification may also explain why ethical leadership contributes to personal growth. Consistent with this reasoning, recent studies suggest that organizational identification plays a central role in connecting ethical or value-based organizational contexts with employee well-being and work outcomes (Barattucci et al., 2021; Onesti, 2023). Therefore, this study proposes the following hypotheses:

*H4a*: Organizational identification plays a mediating role between ethical leadership and positive relations with others.

*H4b*: Organizational identification plays a mediating role between ethical leadership and personal growth.

### The moderating role of caring ethical climate

The effect of ethical leadership may depend on the broader ethical climate in which employees interpret leaders’ behavior. A caring ethical climate emphasizes mutual concern, support, cooperation, and attention to the interests of others (Wei et al., 2019). In such a climate, employees are more likely to perceive ethical leadership as consistent with organizational values rather than as the isolated behavior of an individual leader (Bulutlar, 2025). This consistency between leader behavior and organizational climate can strengthen employees’ trust in the organization and enhance their willingness to identify with it. Prior research suggests that ethical leadership and ethical climate jointly contribute to positive employee outcomes, indicating that leadership effects may become stronger when they are supported by a favorable ethical context (Gwamanda and Mahembe, 2023a). Therefore, a caring ethical climate is expected to strengthen the positive relationship between ethical leadership and organizational identification.

A caring ethical climate may also strengthen the effects of ethical leadership on positive relations with others and personal growth. When ethical leadership is embedded in a climate characterized by care and mutual support, employees are more likely to experience open communication, interpersonal trust, and cooperative interactions. Such an environment can amplify the relational benefits of ethical leadership and help employees build more positive relationships at work (Chen and Tang, 2024). In addition, a caring ethical climate can provide employees with psychological resources and a supportive context for learning, self-development, and autonomous motivation. Prior research has shown that caring ethical climate can function as a boundary condition that strengthens the influence of positive leadership on employee motivation and development-related outcomes (Zhu et al., 2022). Recent research also indicates that organizational ethical climate and ethical leader behavior may jointly shape employees’ value-based responses and positive organizational behavior (Cai et al., 2024). Therefore, this study proposes the following hypotheses:

*H5*: Caring ethical climate positively moderates the relationship between ethical leadership and organizational identification.

*H5a*: Caring ethical climate positively moderates the relationship between ethical leadership and positive relations with others.

*H5b*: Caring ethical climate positively moderates the relationship between ethical leadership and personal growth.

**Figure 1:**
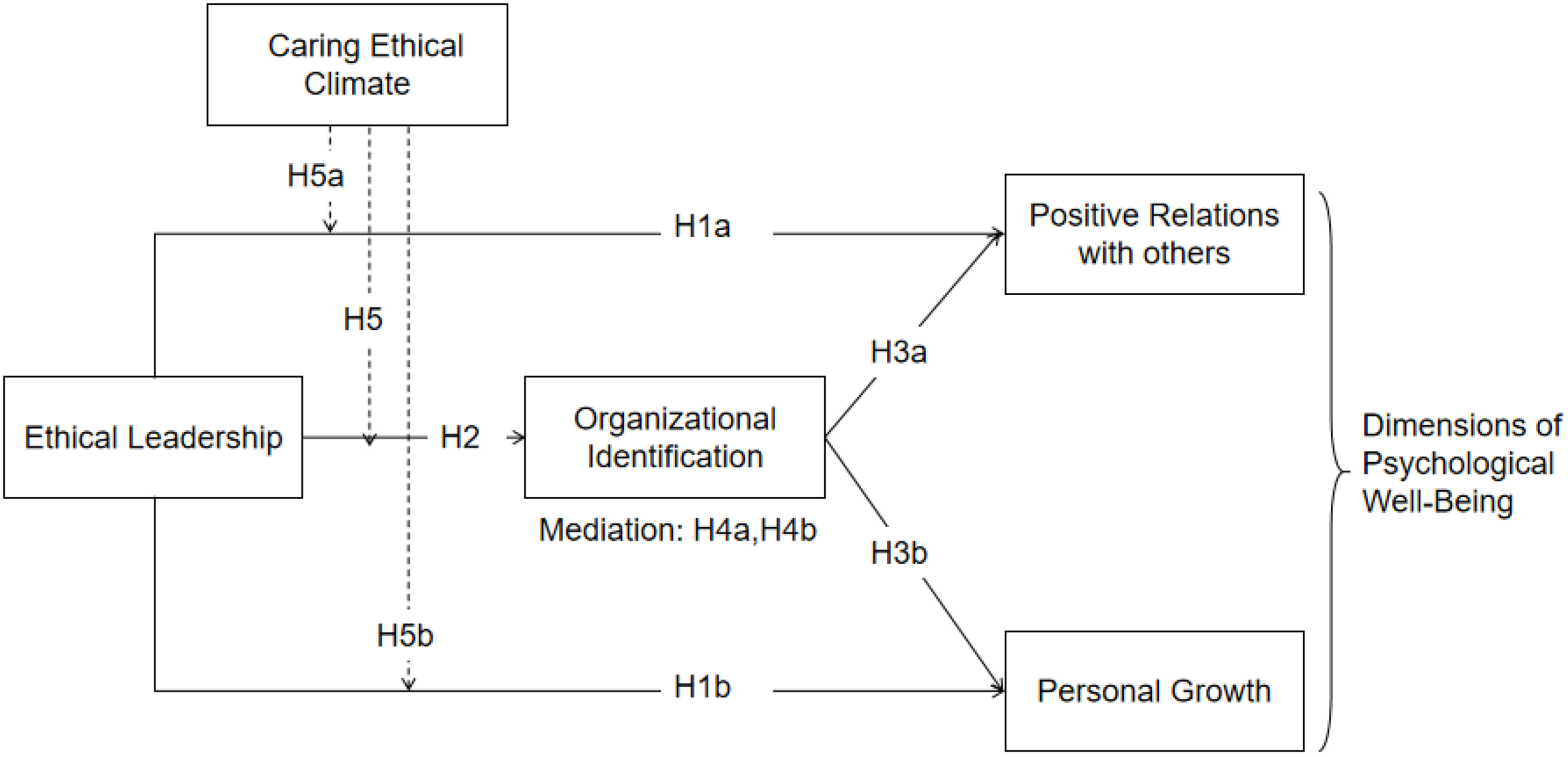
Research model. Solid arrows represent the hypothesized direct effects, and dashed arrows represent the moderating effects of caring ethical climate. Positive relations with others and personal growth are modeled as two dimensions of psychological well-being.

We combined the hypotheses proposed above and formulated a research model, as illustrated in Figure 1.

## Methods

### Data analysis strategy

Confirmatory factor analysis (CFA) was conducted using AMOS 21.0 to assess the measurement model, including reliability, convergent validity, and discriminant validity. The measurement model included five first-order latent constructs: ethical leadership, organizational identification, caring ethical climate, positive relations with others, and personal growth. Positive relations with others and personal growth were treated as two focal dimensions of psychological well-being. After validating the measurement model, structural equation modeling (SEM) was used to test the direct effects and the mediating role of organizational identification. Hierarchical regression analysis was then performed in SPSS 25.0 to examine the moderating role of caring ethical climate. Gender, age, education, position level, and organizational tenure were included as control variables in the regression models.

The measurement model specified five correlated first-order latent constructs: ethical leadership, organizational identification, caring ethical climate, positive relations with others, and personal growth. Each observed item was allowed to load only on its theoretically assigned latent construct, and no cross-loadings were specified. Model fit was evaluated using multiple fit indices, including *χ*^2^/df, CFI, TLI, IFI, RMSEA, and SRMR. Following commonly used criteria, *χ*^2^/df values below 3.00, CFI, TLI, and IFI values close to or above 0.90, and RMSEA and SRMR values below 0.08 were considered acceptable. Reliability and convergent validity were assessed using Cronbach’s *α*, composite reliability, average variance extracted, and standardized factor loadings.

### Sample and procedures

The sample was drawn from regional and national project and property management enterprises in China. These enterprises provide property services, community management, project coordination, customer service, and related operational support. Respondents included frontline staff, frontline managers, and middle managers who were involved in daily service delivery, internal coordination, and customer-facing work. This industry was selected because it is characterized by intensive interpersonal interaction, service pressure, and close coordination across hierarchical levels, which makes ethical leadership particularly relevant to employees’ attitudes and psychological functioning.

Before data collection, the research team contacted enterprise management through professional and academic networks, explained the academic purpose of the study, and obtained permission to distribute anonymous questionnaires. With the assistance of designated contact persons in each enterprise, the survey link was distributed to eligible employees through internal communication channels. Participation was voluntary, and respondents were informed that their answers would be used only for academic research. No personally identifiable information was collected.

To reduce potential common method bias, this study adopted a two-wave time-lagged survey design. The first-wave survey was conducted on December 2, 2025. At T1, 500 questionnaires were distributed, and 400 valid responses were obtained after removing incomplete or invalid questionnaires, yielding a valid response rate of 80.0%. The T1 questionnaire collected demographic information, including gender, age, education, position level, and organizational tenure, as well as employees’ perceptions of ethical leadership and caring ethical climate. Two weeks later, the second-wave survey was administered to the 400 respondents who had provided valid T1 responses. The T2 questionnaire measured organizational identification, positive relations with others, and personal growth. The T2 data collection lasted approximately 1 month, and 310 valid matched questionnaires were finally retained for analysis. The final matched response rate was 77.5% of the T1 valid sample and 62.0% of the initially distributed questionnaires.

Although the sample was not a random national sample of all employees in China’s project and property management sector, it included respondents from different position levels, age groups, educational backgrounds, and organizational tenure groups. Therefore, the sample was considered appropriate for examining the proposed relationships in this specific service-intensive context. Nevertheless, the generalizability of the findings to other industries or cultural contexts should be interpreted with caution.

### Sample characteristics

The final matched sample consisted of 310 valid responses. Among the respondents, 171 were male (55.2%) and 139 were female (44.8%). In terms of age, the largest group was aged 30–39 years (49.4%), followed by those aged 20–29 years (23.5%). Respondents aged 40–49, 50–59, and 60–65 years accounted for 15.8, 9.7, and 1.6% of the sample, respectively. Regarding educational background, most respondents held a junior college degree (38.1%), followed by those with a high school or junior technical secondary school education (32.9%) and those with a bachelor’s degree (28.4%). Only two respondents (0.6%) held a master’s degree or above. In terms of position level, employees and frontline managers accounted for the largest proportion (65.8%), followed by middle managers (28.4%) and senior managers (5.8%). Regarding organizational tenure, most respondents had less than 3 years of work experience (64.5%), followed by those with 4–6 years (21.3%), 7–9 years (8.4%), and more than 10 years (5.8%).

### Measures

This study employed measurement scales derived from well-established instruments validated in previous empirical research. Chinese versions of the scales were developed using translation and back-translation procedures to ensure consistency with the original English items (Brislin, 1980). Apart from demographic variables, all items were assessed using a 7-point Likert scale ranging from 1 (strongly disagree) to 7 (strongly agree). In this study, psychological well-being was operationalized through two focal dimensions: positive relations with others and personal growth.

Ethical Leadership (EL) was measured using the Ethical Leadership Scale originally developed by Brown et al. (2005), which has also been validated and applied in recent organizational and healthcare studies (Zappalà and Toscano, 2020). Sample items include: “My supervisor listens to what others have to say,” “My supervisor makes fair decisions,” and “My supervisor disciplines employees who violate ethical standards.”

Organizational Identification (OI) was measured using items adapted from the organizational identification scale developed by Mael and Ashforth (1992), which remains widely used in recent organizational research (Salin et al., 2023). Sample items include: “When someone criticizes my organization, it feels like a personal insult,” “I identify strongly with my organization’s values,” and “When someone praises my organization, it feels like a personal compliment.”

Caring Ethical Climate (CEC) was measured using items adapted from the Ethical Climate Questionnaire developed by Victor and Cullen (1988). Recent research has further supported the reliability and construct validity of the Ethical Climate Questionnaire in organizational settings (Gwamanda and Mahembe, 2023b). Sample items include: “In this organization, people look out for each other’ s good,” “The organization considers what is best for everyone,” and “People here are genuinely concerned about each other’s welfare.”

Positive Relations with Others (PR) was measured using items adapted from the positive relations with others subscale of Ryff’s Psychological Well-Being Scales (Ryff, 1989; Ryff and Keyes, 1995). This dimension captures the extent to which employees experience warm, trusting, and supportive interpersonal relationships at work. Recent studies on eudaimonic well-being have continued to treat positive relations as a core dimension of psychological well-being (Soren and Ryff, 2023; Carmona-Rincón et al., 2023). Sample items include: “I have warm and trusting relationships with others,” “I know that I can trust my colleagues and friends,” and “I feel supported by people around me.”

Personal Growth (PG) was measured using items adapted from the personal growth subscale of Ryff’s Psychological Well-Being Scales (Ryff, 1989; Ryff and Keyes, 1995). This dimension reflects employees’ perceived continuous learning, self-development, and realization of personal potential. Recent workplace well-being research has also emphasized personal growth as an important aspect of eudaimonic psychological functioning (Soren and Ryff, 2023; Carmona-Rincón et al., 2023). Sample items include: “I continue to learn more about myself as time goes by,” “I see myself as growing and developing,” and “I think it is important to have new experiences that challenge how I think about myself and the world.”

### Common method bias control

To reduce the potential risk of common method bias, this study adopted both procedural and statistical remedies. Procedurally, a two-wave time-lagged survey design was used: ethical leadership, caring ethical climate, and demographic variables were measured at T1, whereas organizational identification, positive relations with others, and personal growth were measured at T2. All questionnaires were completed anonymously, and no personally identifiable information was collected. Statistically, Harman’s single-factor test was conducted. The first factor accounted for 40.67% of the total variance, which was below the 50% threshold, indicating that common method bias was not a serious concern. However, because all substantive variables were measured using self-report questionnaires, common method bias and perceptual consistency bias cannot be completely ruled out.

## Empirical results

Before testing the structural relationships, a confirmatory factor analysis was conducted to evaluate the hypothesized five-factor measurement model. The model included ethical leadership, organizational identification, caring ethical climate, positive relations with others, and personal growth as five distinct but correlated latent constructs. The reliability and convergent validity of the measurement model were then assessed using standardized factor loadings, Cronbach’s *α*, composite reliability (CR), and average variance extracted (AVE). As shown in Table 1, all standardized factor loadings ranged from 0.56 to 0.91, exceeding the recommended threshold of 0.50. The Cronbach’s α values ranged from 0.814 to 0.892, indicating satisfactory internal consistency. The CR values ranged from 0.788 to 0.938, all above the recommended threshold of 0.70. In addition, the AVE values ranged from 0.500 to 0.657, meeting the commonly accepted threshold of 0.50. These results indicate that the measurement scales demonstrated acceptable reliability and convergent validity.

**Table 1:** Reliability and convergent validity results.

Item	Std. loading	Cronbach’s α	CR	AVE
EL3	0.70	0.892	0.938	0.604
EL6	0.86			
EL7	0.78			
EL9	0.76			
EL10	0.88			
EL1	0.72			
EL2	0.75			
EL4	0.77			
EL5	0.79			
EL8	0.74			
PR1	0.70	0.883	0.788	0.554
PR2	0.73			
PR3	0.80			
PG1	0.78	0.875	0.851	0.657
PG2	0.82			
PG3	0.83			
OI3	0.69	0.843	0.877	0.595
OI4	0.91			
OI5	0.90			
OI1	0.56			
OI2	0.74			
CEC1	0.73	0.814	0.833	0.500
CEC3	0.77			
CEC4	0.65			
CEC5	0.67			
CEC2	0.71			

Model fit:*χ*^2^ = 532.836, df = 199, *χ*^2^/df = 2.678, TLI, 0.903; CFI, 0.916; RMSEA, 0.074; SRMR, 0.061; NFI, 0.874; IFI, 0.853.

EL, ethical leadership; PR, positive relations with others; PG, personal growth; OI, organizational identification; CEC, caring ethical climate.

To provide additional evidence for discriminant validity, we compared the hypothesized five-factor model with several alternative measurement models. The alternative models combined theoretically related constructs, including a four-factor model in which positive relations with others and personal growth were combined, a three-factor model in which organizational identification, positive relations with others, and personal growth were combined, a two-factor model in which ethical leadership and caring ethical climate were combined and the three employee outcome-related constructs were combined, and a one-factor model in which all items were loaded onto a single factor. As shown in Table 2, the hypothesized five-factor model showed the best fit among all tested models, providing further support for the distinctiveness of the five constructs.

**Table 2:** Comparison of alternative measurement models.

Description	*χ*^2^	df	*χ*^2^/df	CFI	TLI	IFI	RMSEA	SRMR
EL, OI, CEC, PR, and PG modeled separately	532.836	199	2.678	0.916	0.903	0.853	0.074	0.061
PR and PG combined	698.726	203	3.442	0.832	0.805	0.833	0.089	0.073
OI, PR, and PG combined	975.204	206	4.734	0.814	0.78	0.815	0.11	0.086
EL and CEC combined; OI, PR, and PG combined	1074.32	208	5.165	0.731	0.664	0.732	0.116	0.089
All items loaded onto one factor	1288.067	209	6.163	0.597	0.501	0.598	0.129	0.113

EL, ethical leadership; OI, organizational identification; CEC, caring ethical climate; PR, positive relations with others; PG, personal growth.

Descriptive statistics, correlations, and discriminant validity were examined together. As shown in Table 3, all constructs were significantly and positively correlated, with correlation coefficients ranging from 0.355 to 0.620. These results provide preliminary support for the hypothesized relationships among ethical leadership, organizational identification, caring ethical climate, positive relations with others, and personal growth. Discriminant validity was assessed using the Fornell–Larcker criterion. The square roots of the AVE values, shown on the diagonal, were greater than the corresponding inter-construct correlations, indicating acceptable discriminant validity among the five constructs.

**Table 3:** Descriptive statistics, correlations, and discriminant validity.

Variable	Mean	SD	EL	OI	CEC	PR	PG
EL	5.94	1.01	**0.777**				
OI	5.97	0.93	0.466**	**0.771**			
CEC	5.35	1.02	0.525**	0.455**	**0.707**		
PR	5.56	0.87	0.355**	0.524**	0.432**	**0.744**	
PG	5.74	0.91	0.462**	0.620**	0.579**	0.577**	**0.810**

EL, ethical leadership; OI, organizational identification; CEC, caring ethical climate; PR, positive relations with others; PG, personal growth. Diagonal values in bold italics represent the square roots of AVE. **p < 0.01.

After confirming the reliability and validity of the measurement model, structural equation modeling was used to test the hypothesized direct relationships among ethical leadership, organizational identification, positive relations with others, and personal growth. Because positive relations with others and personal growth were treated as two focal dimensions of psychological well-being, psychological well-being was not modeled as a separate dependent variable in the structural model.

The structural model showed an acceptable fit to the data: *χ*^2^/df = 2.381, GFI = 0.908, AGFI = 0.877, IFI = 0.950, TLI = 0.940, CFI = 0.950, NFI = 0.917, and RMSEA = 0.067. The results of the path analysis are presented in Table 4. Ethical leadership had a significant positive effect on organizational identification (*β* = 0.523, *p* < 0.001), supporting Hypothesis 2. Ethical leadership also had significant positive effects on positive relations with others (*β* = 0.140, *p* < 0.05) and personal growth (*β* = 0.222, *p* < 0.001), supporting Hypotheses 1a and 1b. In addition, organizational identification had significant positive effects on positive relations with others (*β* = 0.529, *p* < 0.001) and personal growth (*β* = 0.586, *p* < 0.001), supporting Hypotheses 3a and 3b.

**Table 4:** Structural path analysis results.

Path	Estimate	S.E.	C.R.	*p*
EL → OI	0.523	0.060	6.823	***
EL → PR	0.140	0.056	2.042	0.041
EL → PG	0.222	0.056	3.805	***
OI → PR	0.529	0.092	6.094	***
OI → PG	0.586	0.100	7.679	***

EL, ethical leadership; OI, organizational identification; PR, positive relations with others; PG, personal growth. S.E., standard error; C.R., critical ratio. *p < 0.05; **p < 0.01; ***p < 0.001.

The mediating role of organizational identification was tested using the bias-corrected bootstrap method with 2,000 resamples. Since positive relations with others and personal growth were treated as two dimensions of eudaimonic well-being at work, the mediation analysis focused on the indirect associations between ethical leadership and these two outcomes through organizational identification.

As shown in Table 5, the indirect association between ethical leadership and positive relations with others through organizational identification was significant (indirect effect = 0.277, *p* < 0.01), with a 95% bias-corrected confidence interval of [0.189, 0.391], excluding zero. This result indicates that organizational identification served as a mediating mechanism in the relationship between ethical leadership and positive relations with others. Since the direct association between ethical leadership and positive relations with others remained significant, Hypothesis 4a was supported, indicating a partial mediation effect.

**Table 5:** Mediation analysis results.

Model path	Estimated effect	Lower bound	Upper bound	Result
EL → OI → PR	0.277**	0.189	0.391	Partial mediation
EL → OI	0.523**	0.390	0.647	
EL → PR	0.140*	0.026	0.293	
OI → PR	0.529**	0.378	0.800	
EL → OI → PG	0.307***	0.277	0.409	Partial mediation
EL → OI	0.523**	0.390	0.647	
EL → PG	0.222***	0.073	0.372	
OI → PG	0.560**	0.440	0.716	

EL, ethical leadership; OI, organizational identification; PR, positive relations with others; PG, personal growth. Bootstrap resampling, 2,000. CI, confidence interval. Mediation is significant when the 95% bias-corrected confidence interval does not include zero. *p < 0.05; **p < 0.01; ***p < 0.001.

The indirect association between ethical leadership and personal growth through organizational identification was also significant (indirect effect = 0.307, *p* < 0.001), with a 95% bias-corrected confidence interval of [0.277, 0.409], excluding zero. This finding indicates that organizational identification served as a mediating mechanism in the relationship between ethical leadership and personal growth. Since the direct association between ethical leadership and personal growth remained significant, Hypothesis 4b was also supported, indicating a partial mediation effect.

Overall, the mediation results suggest that ethical leadership was associated with employees’ positive relations with others and personal growth both directly and indirectly through organizational identification.

Hierarchical regression analysis was conducted to examine the moderating role of caring ethical climate. Ethical leadership and caring ethical climate were mean-centered before creating the interaction terms. As shown in Table 6, the interaction between ethical leadership and caring ethical climate had a significant positive effect on organizational identification (*β* = 0.071, *p* < 0.05), indicating that caring ethical climate positively moderated the relationship between ethical leadership and organizational identification. Thus, Hypothesis 5 was supported.

**Table 6:** Summary of regression analysis.

Variable	OI Model 1	OI Model 2	PR Model 3	PR Model 4	PG Model 5	PG Model 6
EL	0.295**	0.253***	0.183*	0.199*	0.223***	0.170*
CEC	0.246**	0.256**	0.325***	0.321***	0.449***	0.462***
EL × CEC	—	0.071*	—	0.067*	—	0.089*
*R*^2^	0.266	0.276	0.206	0.207	0.364	0.377
Adjusted *R*^2^	0.261	0.269	0.201	0.199	0.36	0.371
*F*	55.69**	38.92***	39.73***	26.59**	87.861***	61.79*
Δ*R*^2^	—	0.01	—	0.011	—	0.013
ΔF	—	4.617***	—	4.461**	—	6.507*
VIF		1.174		1.174		1.174

EL, ethical leadership; CEC, caring ethical climate; OI, organizational identification; PR, positive relations with others; PG, personal growth. Interaction terms were created after mean-centering the predictor and moderator. *p < 0.05; **p < 0.01; ***p < 0.001.

The interaction between ethical leadership and caring ethical climate also had a significant positive effect on positive relations with others (*β* = 0.067, *p* < 0.05), suggesting that the positive relationship between ethical leadership and positive relations with others became stronger when caring ethical climate was higher. Therefore, Hypothesis 5a was supported. In addition, the interaction term had a significant positive effect on personal growth (*β* = 0.089, *p* < 0.05), indicating that caring ethical climate strengthened the relationship between ethical leadership and personal growth. Thus, Hypothesis 5b was supported.

**Figure 2:**
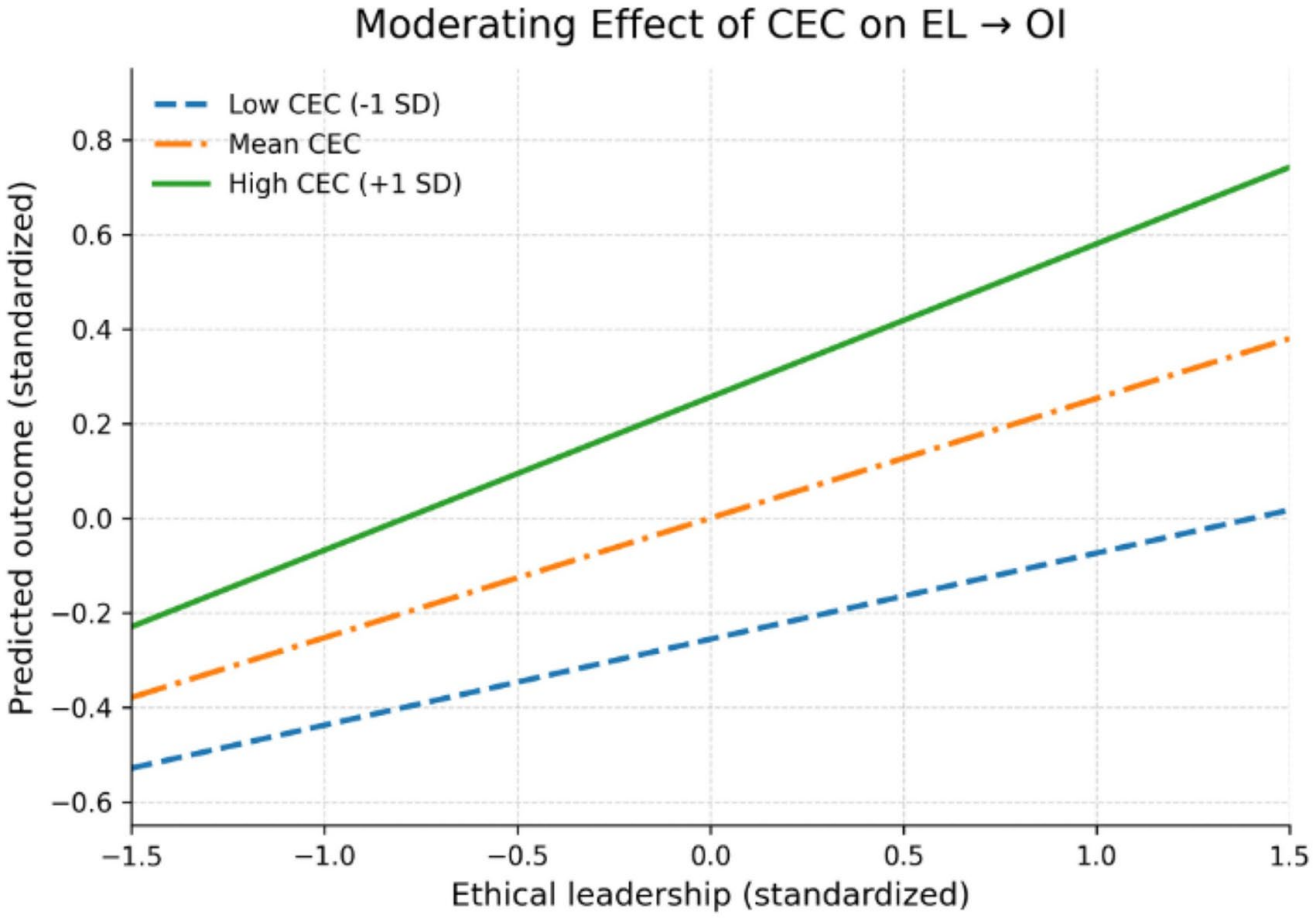
Moderating effect of CEC between EL and OI.

**Figure 3:**
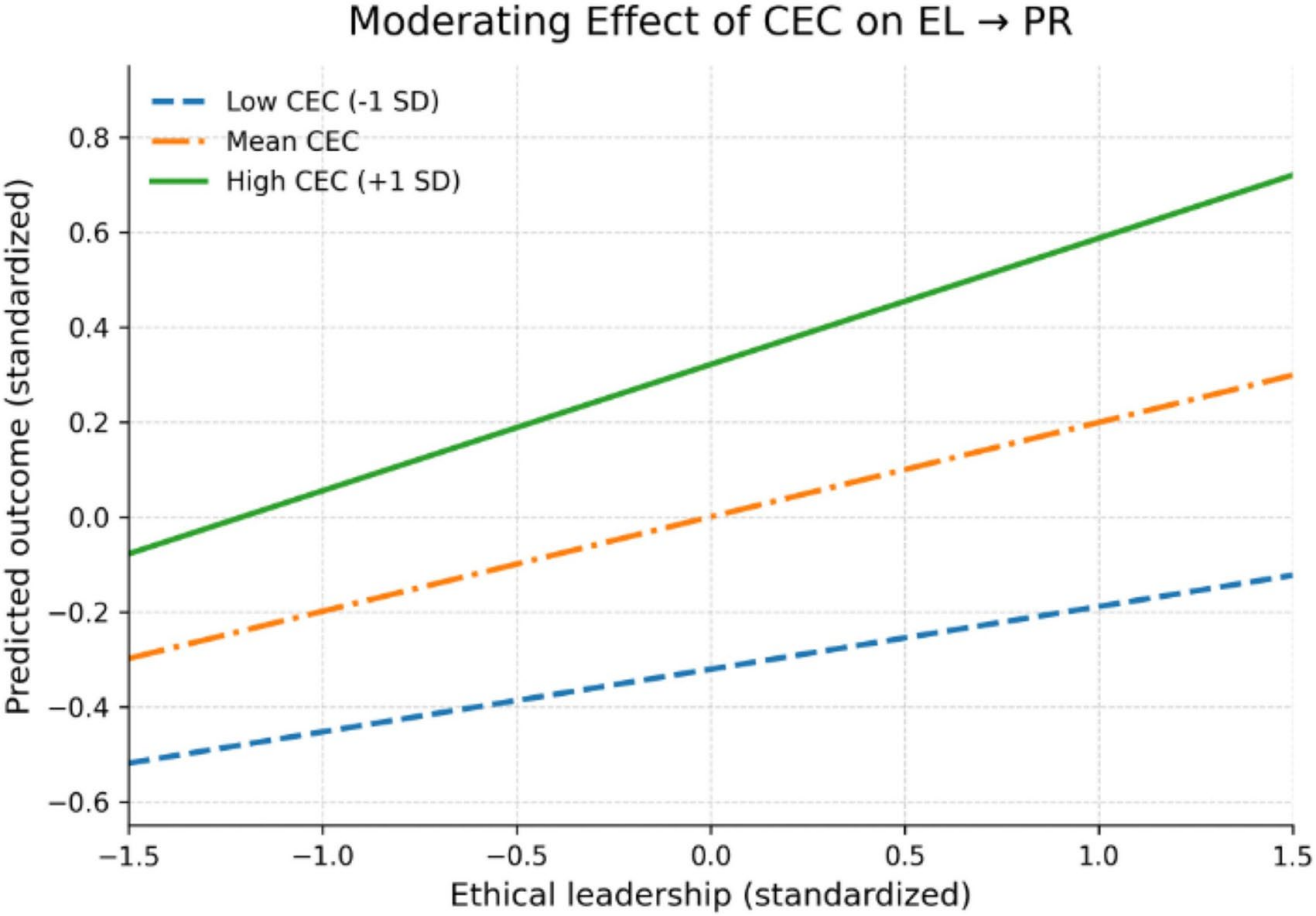
Moderating effect of CEC between EL and PR.

**Figure 4:**
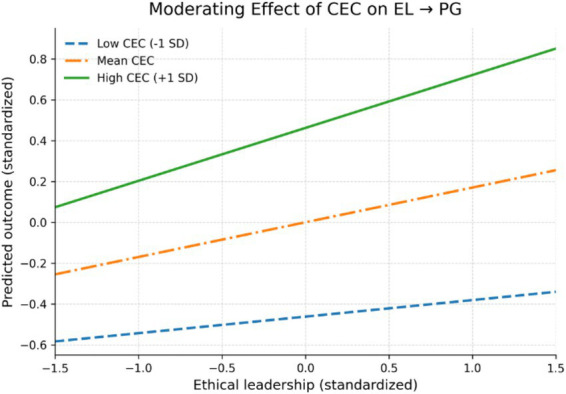
Moderating effect of CEC between EL and PG.

To further interpret the significant interaction effects, simple slope analyses were conducted. As shown in Figure 2, the positive effect of ethical leadership on organizational identification was stronger under high caring ethical climate than under low caring ethical climate. Figure 3 shows that the relationship between ethical leadership and positive relations with others was also stronger when caring ethical climate was high. Similarly, Figure 4 indicates that ethical leadership had a stronger positive effect on personal growth under high caring ethical climate. Overall, these results suggest that caring ethical climate may modestly strengthen the associations between ethical leadership and employees’ organizational identification, positive relations with others, and personal growth. However, because the interaction effects were relatively small, the moderating role of caring ethical climate should be interpreted as incremental rather than decisive.

## Discussion and conclusion

### Discussion

This study examined how ethical leadership contributes to employees’ psychological well-being in project and property management enterprises in China. Rather than treating psychological well-being as a single broad outcome, this study focused on two specific dimensions: positive relations with others and personal growth. The results provide empirical support for the proposed mediation and moderation model and suggest that ethical leadership is associated with employees’ relational and developmental well-being through both direct and indirect associations.

The findings can be explained from an identity-based perspective. Ethical leaders provide employees with fairness, care, moral consistency, and trustworthy interpersonal treatment. These behaviors may help employees perceive the organization as a fair and supportive social environment, thereby strengthening their organizational identification (Brown et al., 2005). When employees identify with their organization, they are more likely to experience belonging, value congruence, and meaningful connection with the work environment. These psychological experiences may support more positive interpersonal relationships and stronger personal growth. Thus, organizational identification serves as an identity-based pathway linking ethical leadership to employees’ relational and developmental well-being (Hameed et al., 2022).

The moderating role of caring ethical climate provides further contextual explanation. Because a caring ethical climate emphasizes mutual care, support, and concern for collective welfare, employees are more likely to interpret ethical leadership as consistent with broader organizational values (Kuenzi et al., 2020). In such a context, ethical leadership may be more easily recognized, trusted, and internalized by employees, thereby strengthening its associations with organizational identification, positive relations with others, and personal growth. However, because the interaction effects were relatively small, caring ethical climate should be interpreted as an incremental boundary condition rather than a decisive factor.

### Implications for management

The findings of this study provide practical implications for managers in project and property management enterprises. Since ethical leadership was found to promote employees’ positive relations with others and personal growth, organizations should regard ethical leadership as an important managerial capability rather than only a moral expectation (Mayer et al., 2012). Managers should demonstrate fairness, honesty, care, and consistency in daily decision-making, communication, performance evaluation, and conflict resolution. In service-intensive organizations, employees frequently face customer complaints, coordination pressure, and interpersonal conflicts. Under these conditions, ethical leaders can reduce uncertainty by clarifying acceptable conduct, treating employees with respect, and providing stable behavioral standards. Such practices can help employees build trust-based relationships and maintain a more positive psychological state at work.

The results also suggest that organizational identification is an important pathway through which ethical leadership enhances employees’ psychological well-being. Therefore, managers should strengthen employees’ sense of belonging and value alignment with the organization. This can be achieved by clearly communicating organizational values, explaining the meaning of employees’ work, and showing how individual contributions support broader organizational goals. When employees perceive that their organization is fair, trustworthy, and consistent with their own values, they are more likely to identify with the organization and transform this identification into cooperative behavior, supportive interpersonal relationships, and personal development. For project and property management enterprises, this is particularly important because employees often work in boundary-spanning roles that require continuous interaction with residents, clients, contractors, and internal departments.

The moderating effect of caring ethical climate further indicates that ethical leadership is more effective when it is supported by a broader organizational environment characterized by care, mutual support, and concern for collective welfare. Organizations should therefore avoid relying only on individual leaders’ ethical behavior and should build ethical practices into daily management systems. For example, enterprises can establish fair complaint-handling procedures, transparent communication channels, supportive team routines, and mechanisms for reporting ethical concerns without fear of retaliation. These practices can help employees perceive ethical leadership as part of the organization’s overall value system rather than as isolated behavior by individual supervisors.

Taken together, the findings imply that employee psychological well-being can be strengthened through the coordinated development of ethical leadership, organizational identification, and caring ethical climate. Managers should not treat employee well-being as a separate welfare issue detached from leadership and organizational culture. Instead, ethical leadership training, value-based communication, employee participation, and climate-building practices should be integrated into human resource management and daily operations. By doing so, organizations can create a work environment in which employees are more likely to develop positive relationships, experience personal growth, and contribute to sustainable service performance.

### Research limitations and future research

This study has several limitations that should be considered when interpreting the findings. Although this study adopted a two-wave time-lagged design, all substantive variables were measured using self-report questionnaires. Therefore, common method bias and perceptual consistency bias cannot be completely ruled out. Moreover, the time-lagged design helped establish temporal separation among the variables but did not allow strong causal conclusions. Future research could adopt longitudinal, experimental, or multi-source designs, such as supervisor ratings, peer evaluations, or objective performance indicators, to provide stronger evidence for the directionality of the proposed relationships. In addition, the sample was drawn from project and property management enterprises in China, which limits the generalizability of the findings to other industries or cultural contexts. Future research could examine whether the proposed model applies to other service sectors, manufacturing firms, public organizations, or cross-cultural samples.

This study focused on positive relations with others and personal growth as two dimensions of psychological well-being. Although these dimensions are theoretically relevant to the research context, future studies could include other dimensions of psychological well-being, such as autonomy, purpose in life, environmental mastery, or self-acceptance, to provide a more comprehensive understanding of employee well-being. The current model examined organizational identification as the mediating mechanism and caring ethical climate as the boundary condition. Future research could further explore other psychological mechanisms, such as psychological safety, work meaningfulness, trust in leader, or perceived organizational support. Additional moderators, such as job insecurity, work pressure, or leadership distance, may also help explain when ethical leadership is more or less effective in promoting employee well-being.

## Conclusion

This study contributes to the ethical leadership and employee well-being literature by showing that ethical leadership is associated with two dimensions of eudaimonic well-being at work: positive relations with others and personal growth. The findings indicate that organizational identification partially mediates these relationships, while caring ethical climate strengthens the associations between ethical leadership and employee outcomes. Overall, this study suggests that employees’ relational and developmental well-being is shaped not only by leadership behavior, but also by identity-based psychological mechanisms and the broader ethical climate of the organization.

## Data Availability

The original contributions presented in the study are included in the article/supplementary material, further inquiries can be directed to the corresponding authors.
